# Editorial: Application of Neural Technology to Neuro-Management and Neuro-Marketing

**DOI:** 10.3389/fnins.2020.00053

**Published:** 2020-02-14

**Authors:** Ioan Opris, Sorin Cristian Ionescu, Mikhail A. Lebedev, Frederic Boy, Peter Lewinski, Laura Ballerini

**Affiliations:** ^1^Department of Biomedical Engineering, University of Miami, Coral Gables, FL, United States; ^2^Faculty of Entrepreneurship, Business Engineering and Management, Politehnica University, Bucharest, Romania; ^3^Duke Center for Neuroengineering, Duke University, Durham, NC, United States; ^4^Center for Bioelectric Interfaces of the Institute for Cognitive Neuroscience, National Research University Higher School of Economics, Moscow, Russia; ^5^Department of Information and Internet Technologies of Digital Health Institute, I.M. Sechenov First Moscow State Medical University, Moscow, Russia; ^6^Innovation Lab (iLab), Department of Business, School of Management, Swansea University, Swansea, United Kingdom; ^7^Department of Medical Physics and Biomedical Engineering, University College London (UCL), London, United Kingdom; ^8^Saïd Business School, University of Oxford, Oxford, United Kingdom; ^9^Neuroscience Area, International School for Advanced Studies (SISSA), Trieste, Italy

**Keywords:** neuromarketing, neuromanagement, brand preference, consumer, ERPs

Marketing studies the management of exchange relationships, while brand management deals with the relationship between a company's product and emotional perception of customer in terms of expectations and satisfaction. Here we are set to assess work on neuro-management and neuro-marketing by investigating the neural features of customer/consumer behavior.

## Brand Management

Several reports describe the brand management in terms of brand preferences, brand extension, personalized preference, perception and framing of price, and hazard perception (in term of financial decision making).

### Brand Preference

The preference for a certain brand in the context of brand competition is a sign of customer loyalty. Yu et al. examined brand preference rating by recording ERPs to analyze brain activity during brand information processing when subjects/customers experienced victory or defeat. Behavioral results showed that subjects exhibited a stronger preference for “unfamiliar” brands in victory trials, even if the brand was totally unrelated to the competition. The existence of “incidental emotions” induced by victory or defeat were reflected in the elicited ERPs, more negatively in victory than in defeat trials. These findings indicate that victory/defeat contexts can evoke “incidental emotions” that induce preference for unfamiliar brands.

The customers' preference is biased to their local brands over the foreign ones. This tendency is known was coined as consumer ethnocentrism. Ma, Abdeljelil et al. employed the event-related potential (ERP) approach to identify consumer ethnocentrism on brand preference in terms of neural activity and behavioral responses on two groups of subjects. One group consisted of Chinese subjects and the other group was formed by Black Africans. The results pointed out that the race significantly impacted the Chinese subjects' brand preference. There was also evidence that familiarity with foreign cultures reduces consumer's ethnocentrism. African subjects being familiar with Chinese people had similar brand preferences.

### Brand Extension

Brand extension is a marketing approach in which a new product such as goods or service is launched under an established brand name. It distinguishes two strategies: brand name extension (BN) and brand logo extension (BL). Shang et al. assessed which of the two strategies (BN or BL) better increased the success of dissimilar brand extension. Behavioral outcomes demonstrated that BL was accepted better than BN in the dissimilar brand extension. At the neurophysiological level, the brand extension process was characterized by a less negative N2 and a larger P300 in the BL compared to BN. Yang and Kim compared ERPs between population-fit groups and found significant differences in the fronto-central N2 and fronto-parietal P300 amplitudes. Their findings indicate that left fronto-parietal P300 may yield the evidence for brand extension to service, which may require retrieval of semantic memory and categorization of similarity. Similarly, Yang T. et al. reported ERP analysis that identified three components during the evaluation of brand extension: N2, P300, and N400. Shang et al. indicated that N2 reflected a conflict between the brand-product combination and the long-term memory and that P300 could be regarded as the reflection of the categorization process in the working memory.

### Personalized Preference

Neuroscientific methods are preferred to gain insight into the neural basis of consumers' evaluation of experience good designs. Ma Y. et al. used “personalized” T-shirt designs as stimuli, while recording ERPs in a modified go/no-go task to investigate consumers' neural responses to experience good designs. Results show that both ERP components, P200 and the late positive potential (LPP), were high in response to the best-preferred product designs vs. the least-preferred designs, when subjects saw the product designs without making a decision. These findings shed light on the reasons why consumers like customized products.

### Price: Perception and Framing

Two studies are reporting on price perception and the temptation of zero price, while providing evidence on price framing in purchase decision making Emotion was demonstrated to be ubiquitous in marketing and influenced purchase processing as well. The study by Ma, Zhang et al. examined whether emotion arousal would influence consumers' price perceptions and their willingness to purchase. Both behavioral and ERP results indicated that subjects' price perception was deeply impacted by emotions induced from continuous win/lose experiences.

Ma H. et al. employed the ERPs approach to investigate the role of “price framing” in information processing and purchase decision making in a bundling context. Results provide both behavioral and neural evidence for how different price framing information is processed and ultimately gives rise to price framing effect in purchase decision making.

### Hazard Perception and Financial Decision Making

A couple of papers report on the “hazard perception” for the warning signs and on the sequential organization of information for financial decision-making. Ma, Bai et al. used ERP technology and the “Oddball paradigm” to evaluate the impact of the shapes on the perception of warning signs, and to discover the neural substrate of the “hazard perception” of the shapes by electrophysiological characterization. Results indicated that the shape of “upright triangle” provides larger arousal intensity and more “negative valence” than the shape of “circle.” Stronger negative information comes from the “upright triangle” shapes than from the “circle.” This finding may be useful for designing the shapes surrounding warning signs.

Yang W. et al. have evaluated the case when the evidence and information are in a sequence and have found that order effect and biases have an impact in various areas. The behavioral outcomes, which are an investment decision, were consistent with the idea that individuals will invest more/retire less when receiving the information in a negative-positive order. The results suggest that in the scheme that involve large-scale information, the organization of information (integration vs. segregation) influences the emotion and approach-withdraw trend. Deppe et al. (2005) further add that nonlinear responses within the medial prefrontal cortex reveal when specific implicit information is influencing the process of economic decision making.

## Use of Neural Technology in Marketing Research

The reports below describe the application of the neural technology in marketing research in combination with: fNRI, lie detection, advertisement research, fake rating behavior in e-commerce and stereotypes activated by media.

### fNIRS in Marketing Research

Krampe et al. investigated—likely for the first time ever- the validity of the mobile functional near-infrared spectroscopy (fNIRS) in (neuro-) marketing research. Successfully, the authors managed to replicate the second sub-effect (reduced activity in dlPFC) but as they hypothesized, they failed to replicate the first sub-effect (reduced activity in vmPFC) of the “*first-choice-brand*” effect (Deppe et al., 2005) using mobile fNIRS. Therefore, it might be concluded that if neuromarketing researchers are interested in measuring activity in dlPFC, mobile fNIRS offers them more affordable and ecologically valid tool than fMRI, at least while measuring the “first-choice-brand” effect.

### Lie Detection

Growing interest and importance of the fNIRS is additionally strengthened by Li F. et al. (this Research Topic) study of a topic inextricably linked to (neuro-) management—detection of infrequent and frequent liars. The authors specifically found that “while performing deception detection tasks, infrequent liars showed significantly greater neural activation in the left MFG than the baseline, but frequent liars and innocents did not exhibit this pattern of neural activation in any area of inhibition-related brain regions” (p. 1). Li D. et al.'s findings and application of fNIRS—during for example prisoner's dilemma—might shed further light on that cornerstone of the game theory and modern management decision-making and further unite neuroscience and management fields.

### Advertisement Research

Several papers discuss topics of modern advertisement research involving novel technology based on support vector machine for impact assessment, willingness to pay reflected in brain activity, fake rating in e-commerce, and racial stereotypes activated by media.

The current Research Topic reports two studies which focus on application of electroencephalography (EEG) to the advertisement research. Ramsøy et al. established a relation between widely studied willingness to pay (WTP) measure and EEG responses: the prefrontal gamma asymmetry and a trend in the beta frequency band. Then, importantly, Wei et al. developed—based on data from 220 different video advertisements—a novel artificial intelligence algorithm (Support Vector Machine; SVM) to predict WTP (just like Ramsøy et al.) by using neural responses but from a consumer-grade (and hence low-cost) EEG headset; with an accuracy of around 75%. Taken together, these studies significantly advance the study of neuromarketing.

### Fake Rating Behavior in E-Commerce

In a study on e-commerce, Wang et al. explored whether a certain strategy was more likely to give rise to false rating behaviors, as assessed by ERPs. A two-stimulus paradigm was used to show that five-star ratings strategy led to a higher rate than the other. The findings provide evidence supporting the policy of forbidding the use of the “five stars rating” strategy in e-commerce.

### Racial Stereotypes Activated by Media

Stereotypes from the major nationality toward minorities constitute a widely concerning problem in many countries. The study by Jin et al. focused on the neural basis of the modulation of negative media information on Han Chinese stereotypes toward Uyghurs by using ERPs measures. The results suggested that the negative media information might influence their judgments toward other groups reflected in the deflection of N400 amplitude. Therefore, in order to mitigate or even eliminate stereotypes about national minorities, the effort of the media is important.

## Neural Correlates of Neuromarketing

Several reports describe the neural correlates of: monetary incentive delay, self-motivation, preference, consumer buying motivations, reputation learning, the relationship between challenge and motivation, and the neural features of safety inspections.

### Monetary Incentive Delay

Krugliakova et al. investigated whether an auditory version of the monetary incentive delay task could modulate brain plasticity when processing incentive cues that code for expected monetary outcomes. Interestingly, they found that after only 2 days of training, auditory stimuli predicting reward evoked a larger involuntary neural response than that in the baseline condition. Their result suggests that the sensory processing of incentive cues is dynamically adjusted by the expectation of a reward.

### Self-Motivation

Within the framework of the self-determination theory, Fang et al. examined restorative processes occurring when competence, one of the basic psychological predictor of one's wellbeing, is frustrated. To this effect, they manipulated competence frustration in a between-group experimental design, and observed an enlarged frustration-related neural response in the experimental group, as compared to controls. Such results document the neural correlates of complex restoration process and can help refine future managerial practice.

### Neural Correlates of Preference

Akiba et al. explored the physiological markers of likeability judgements, a crucial element in value-based evaluation, the building brick of the reward system. To this effect, they developed a method capable of tracking bodily reactions to dynamic video stimuli and were able to identify robust time-dependent markers of pleasure/displeasure judgements.

### Neural Correlates of Consumer Buying Motivations

In their study, Goodman et al. used functional magnetic resonance imaging (fMRI) to assess and contrast the neural correlates of various types of buyer's motivations for ordinary consumer goods. The results segregated different patterns of activations with symbolic buying motivation associated more with medial frontal gyrus (MFG) BOLD signal, experiential motivation associated more with posterior cingulate cortex (PCC) activation, and functional motivation associated more with activity in the dorsolateral prefrontal cortex (DLPFC). These findings elucidate some of the neural underpinnings of reduced self-control.

### Reputation Learning

In an event-related potential (ERP) study, Li D. et al. explored the reputation learning process in a repeated trust game where subjects made multiple round decisions of investment to partners. They found that subjects gradually learned to discriminate trustworthy partners from untrustworthy ones based on how often their partners reciprocated the investment. In the late stages of the game, this discrimination was matched in the electrophysiology, where the faces of untrustworthy partners induced larger feedback negativity (FN) than those of trustworthy partners. This result highlights the fact that the FN reflects the reputation appraisal and is useful to tracks reputation learning in social interactions.

### Relationship Between Challenge and Motivation

The balance between task demand and one's competence is critical for the maintenance of motivation. Ma, Pei et al. employed the inverted U-shaped curve to depict the relationship between a player's perceived challenge and his motivation. The electrophysiological results confirmed the inverted U-shaped curvilinear relationship between perceived challenge and one's intrinsic motivation.

### Neural Features of Safety Inspections

The study by Ma, Shi et al. used ERPs to investigate the neurocognitive signatures of “severe-and-deterrent phrases” and “mild-and-polite phrases” used by OHS inspectors. The ERP results demonstrated that first type of phrases convey a higher level of severity and motivation compared with the second type of phrases, that exhibited a significantly incremented P300 amplitude. The study by Ma, Shi et al. provides an objective method to quantify the efficiency of “enforcement phrases,” which may help to enhance quality of OHS inspections.

## Author Contributions

All authors listed have made a substantial, direct and intellectual contribution to the work, and approved it for publication.

### Conflict of Interest

The authors declare that the research was conducted in the absence of any commercial or financial relationships that could be construed as a potential conflict of interest.
